# MitoTox: a comprehensive mitochondrial toxicity database

**DOI:** 10.1186/s12859-021-04285-3

**Published:** 2021-07-15

**Authors:** Yu-Te Lin, Ko-Hong Lin, Chi-Jung Huang, An-Chi Wei

**Affiliations:** 1 Graduate Institute of Biomedical Electronics and Bioinformatics, National Taiwan University, Taipei, Taiwan; 2 Department of Electrical Engineering, National Taiwan University, Taipei, Taiwan

**Keywords:** Database, Mitochondria, Mitochondrial toxicity, Toxin-target association

## Abstract

**Background:**

Mitochondria play essential roles in regulating cellular functions. Some drug treatments and molecular interventions have been reported to have off-target effects damaging mitochondria and causing severe side effects. The development of a database for the management of mitochondrial toxicity-related molecules and their targets is important for further analyses.

**Results:**

To correlate chemical, biological and mechanistic information on clinically relevant mitochondria-related toxicity, a comprehensive mitochondrial toxicity database (MitoTox) was developed. MitoTox is an electronic repository that integrates comprehensive information about mitochondria-related toxins and their targets. Information and data related to mitochondrial toxicity originate from various sources, including scientific journals and other electronic databases. These resources were manually verified and extracted into MitoTox. The database currently contains over 1400 small-molecule compounds, 870 mitochondrial targets, and more than 4100  mitochondrial toxin-target associations. Each MitoTox data record contains over 30 fields, including biochemical properties, therapeutic classification, target proteins, toxicological data, mechanistic information, clinical side effects, and references.

**Conclusions:**

MitoTox provides a fully searchable database with links to references and other databases. Potential applications of MitoTox include toxicity classification, prediction, reference and education. MitoTox is available online at http://www.mitotox.org.

## Background

Mitochondria, the so-called “energy powerhouse” of a cell, generate approximately 95% of cellular adenosine triphosphate (ATP) through oxidative phosphorylation. Mitochondria are also essential for life-supporting functions and cell fate decisions. Mitochondria play multiple roles in the regulation of cellular physiological and pathological mechanisms, including ATP generation, metabolic control, signal transduction, immune response, and apoptosis [1, 2]. Mitochondrial function and dynamics may change due to pathological responses during disease development. In many cases of mitochondrial dysfunction, oxidative phosphorylation is inhibited, thus inducing an elevated level of glycolysis, which leads to fatal lactate accumulation in the serum [3]. Other potential syndromes include cardiomyopathy, rhabdomyolysis, peripheral neuropathy, optic neuropathy, and hepatic steatosis, which are often observed in mitochondrial diseases [4]. Mitochondrial dysfunction has been linked to common diseases, such as cardiovascular diseases, neurodegenerative diseases, diabetes, and cancer [5].

Moreover, mitochondria have been recognized as unintended drug targets of many pharmaceutical and therapeutic agents that can damage mitochondria and lead to changes in mitochondrial morphology and function. Among these, drug-induced mitochondrial toxicity was found to be responsible for cardiotoxicity, hepatotoxicity, neurotoxicity, nephrotoxicity, ototoxicity, and many other organs-related toxicities [6–8]. The heart, brain, and liver, which rely heavily on oxidative phosphorylation or serve as the main organ of drug metabolism, appear to be the main targets of mitochondrial toxicity [9]. Since the late 1990s, the US Food and Drug Administration has withdrawn dozens of drugs from the market due to their hepatotoxicity or cardiotoxicity, and many of these drugs have been linked to mitochondrial dysfunction [10]. For example, troglitazone, a member of the thiazolidinedione class of antidiabetic drugs, was withdrawn in 2000 with reported lethal hepatotoxicity caused by the off-target effect on the mitochondrial electron transport chain (complex I) [7, 11]. Cerivastatin, a lipid-lowering drug withdrawn in 2001 due to rhabdomyolysis, has been linked to mitochondrial toxicity by affecting complex III-related respiration [12, 13]. Table 1 lists additional examples of pharmaceutical drugs that are related to mitochondrial toxicity. Attention was directed to the mitochondria because of these severe side effects, and drug development has begun to consider mitochondria-related toxicity as well as mitochondrial-targeted therapeutics [10]. For cardiologists, this is appealing because mitochondria are the major source of ATP and reactive oxygen species (ROS) in the myocardium and because mitochondrial dysfunction plays an important role in heart failure and arrhythmias [14]. Several drugs used in clinical settings have shown cardiac toxicity due to their direct effects on cardiac mitochondria [15]. Understanding how these molecules act on mitochondria can provide a mechanistic explanation for their toxicological or pharmacological effects.

**Table 1:** Examples of mitochondrial toxicity-related drugs in MitoTox

Indication	Drug Name	Mechanism of mitochondrial toxicity	Status	Toxicity	t_1/2_ (h)^a^	Protein binding (%)^a^	LD_50_ (rat)^a^
Antidiabetic	Troglitazone	Inhibition of ETC	Withdrawn, 2000	Hepatotoxicity	16–34	> 99%	1.9768 mol/kg
	Rosiglitazone	Inhibition of ETC	Approved, investigational	Cardiotoxicity	3–4	99.8%	2.4515 mol/kg
	Pioglitazone	Inhibition of ETC	Approved, investigational	Cardiotoxicity	3–7/16–24	> 99%	2.0115 mol/kg
	Ciglitazone	Inhibition of ETC	Discontinued	NA	NA	NA	NA
	Darglitazone	Inhibition of ETC	Discontinued	NA	NA	NA	NA
	Muraglitazar	Inhibition of ETC	Discontinued	NA	NA	NA	NA
	Metformin	Inhibition of complex I; uncoupling; impaired TCA cycle	Approved	Lactic acidosis	6.2	> 90%	1000 mg/kg
Anticancer	Doxorubicin	Increased ROS; mtDNA adduct; iron overload	Approved	Cardiotoxicity	20–48	74–76%	21.8 mg/kg
	Cisplatin	Inhibition of Complex I	Approved	Nephrotoxicity	0.3–0.7	> 90%	2.7612 mol/kg
Hyperlipidemia	Fenofibrate	Inhibition of complex I	Approved	Hepatotoxicity	20	~ 99%	> 2000 mg/kg
	Clofibrate	Inhibition of complex I	Approved	Hepatotoxicity	18–22	95–97%	940 mg/kg
	Ciprofibrate	Inhibition of complex I	Approved	Hepatotoxicity	NA	NA	NA
Psychotropic	Valproic acid	Inhibition of TCA cycle	Approved	Coma and respiratory depression	9–16	90%	670 mg/kg
	Clozapine	Inhibition of the ETC	Approved	Metabolic syndrome	8	97%	3.0838 mol/kg
	Fluoxetine	Uncoupler	Approved	CNS, GI effects	1–3 days	94.5%	2.6048 mol/kg
	Nefazodone	Inhibition of Complex I and complex IV	Withdrawn	Hepatotoxicity	2–4	> 99%	2.9067 mol/kg
Analgesic	Acetaminophen	Oxidative stress	Approved	Hepatotoxicity	1–4	25%	1944 mg/kg
	Aspirin	mPTP potentiation	Approved	Cardiotoxicity, GI effects	0.25	99.5%	920–1480 mg/kg
	Diclofenac	Inhibition of ETC and ATP synthase	Approved	Nephrotoxicity	2	> 99%	3.6447 mol/kg
Antibacterial	Imipenem	Oxidative stress	Approved	Nephrotoxicity	1.3–5.1	20%	1.8089 mol/kg
	Chloramphenicol	Depletion of iron	Approved	Aplastic anemia	1.5–3.5	50–60%	2500 mg/kg
Antiviral	Zidovudine	mtDNA replication (inhibit mtDNA polymerase-γ)	Approved	Myopathy	0.5–2.9	30–38%	NA

^a^From DrugBank (https://www.drugbank.ca/)

In addition to pharmaceutical exposures, environmental factors and pollutants have been identified as previously unrecognized mitochondrial toxicants, including neurotoxin 1-methyl-4phenyl-1, 2, 3, 6-tetrahydropyridine (MPTP), pesticides (e.g., paraquat and rotenone), and heavy metals (e.g., mercury and cadmium); all of which are now widely recognized mitochondrial toxins [16, 17]. Mitochondrial damage has a broader impact on health than is commonly recognized.

Drug-induced mitochondrial toxicity has been studied for more than fifty years. Multiple mechanisms of mitochondrial toxicity have been reported, including inhibition of oxidative phosphorylation, uncoupling, oxidative stress, irreversible opening of the mitochondrial permeability transition pore, inhibition of fatty acid oxidation or TCA cycle to reduce NADH and FADH_2_ production, and impairment of mitochondrial DNA (mtDNA) replication or mtDNA‐encoded protein synthesis [11, 18]. Although large-scale screening studies reporting drug-induced mitochondrial membrane potential loss [19–21], the mechanisms of mitochondrial toxicity and dysfunction are incompletely understood.

A number of public toxicant databases, such as Aggregated Computational Toxicology Resource (ACToR) [22], Toxin and Toxin Target Database (T3DB) [23], and the Comparative Toxicogenomics Database (CTD) [24], provide comprehensive information about the bioassay data, chemical structures of the toxicants, toxin targets, and chemical-gene-disease relationships. The NIH Tox21 project has developed a library of ~ 10,000 chemicals and drugs (Tox21 10 K), and has generated > 50 million quantitative high-throughput screening (qHTS) data points using over 70 cell-based assays covering a broad range of biological pathways. A few studies have described the screening of the effects on mitochondrial membrane potential and other mitochondrial functional indicators using the Tox21 library [19, 21, 25]; however, there is a lack of currently available database specifically dedicated to mitochondrial toxicity or recording of multiple aspects of the toxin-mitochondria relationships. A keyword search of DrugBank, a public database for drug information [26], demonstrated that “mitochondrial” appears in the list of keywords of only 81 drugs. A PubMed search using the keyword “mitochondrial” resulted in approximately 350,000 hits, and the number of hits for a search for “mitochondrial toxicity” was approximately 31,000 (as of August 2020). These statistics imply that mitochondria are an important research topic; however, the attention to mitochondrial toxicity is insufficient.

Limited information about drug-induced mitochondrial toxicity is available in the literature, and there is no public database specifically for mitochondrial toxicity. With the increasing demands for integrating basic and clinical research, the current paper aims to develop an integrative database for mitochondrial toxicity and identify the toxicological mechanism and corresponding mitochondrial targets. Understanding how these molecules alter mitochondrial phenotype can provide a mechanistic explanation for their toxicological or pharmacological effects on the mitochondria, which will facilitate the development of safer and more effective drugs. This open-access database will help develop and improve detection and screening procedures for mitochondrial toxicity and further facilitate the identification and analysis of mitochondrial functional and pathological changes.

## Methods

### Database construction

Many mechanisms cause mitochondrial toxicity, as mentioned previously. Drugs and compounds that have been reported to exhibit mitochondrial toxicity in the literature were first collected into the mitochondrial toxicity database MitoTox. Through literature searches of journal articles and review papers, drugs and compounds with validated evidence of drug-induced mitochondrial toxicity were recorded. The keywords for mitochondrial toxicity were used to search the collected texts to characterize the mechanisms of mitochondrial toxicity of each drug. Chemical properties, bioactivity, and clinical applications, toxicity and side effect information of the compounds are automatically curated in the MitoTox database from the DrugBank, PubChem, UniProt, and SIDER databases. Additional information is curated manually from the KEGG, BRENDA, IPA [27], LiverTox (http://livertox.nih.gov/; search term: “mitochondria”), and MedicinesComplete (AHFS Drug Information, [online] London: Pharmaceutical Press http://www.new.medicinescomplete.com/, search term: “mitochondrial”) databases.

For example, to acquire clinical information on these drugs, side effects, frequency of side effects, indications, and ATC code, files were downloaded and analyzed from the SIDER Database (http://sideeffects.embl.de/) and DrugBank (https://go.drugbank.com/atc). The drug synonym names and drug targets from the “external Drug Links” and “target Drug-UniProt Links” were obtained from the DrugBank website. To further expand our drug list, the complete DrugBank database XML file (version 5.1.4) was downloaded and manually curated from the DrugBank database (https://www.drugbank.ca/releases/latest) to perform a further search of the text relevant to mitochondria. The Ingenuity Pathway Analysis (IPA; QIAGEN Inc., https://www.qiagenbioinformatics.com/products/ingenuity-pathway-analysis) database (as of Novmember 2020; version 57662101) was also used to find the correlation of mitochondrial function and target molecules.

### Database implementation

**Fig. 1:**
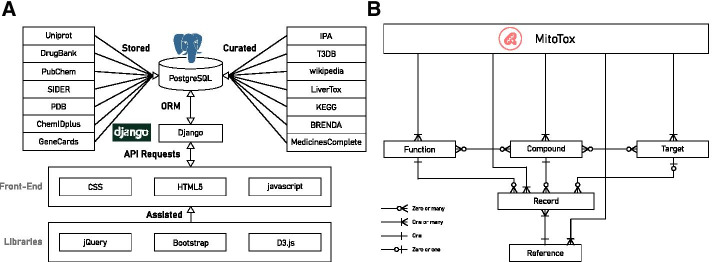
MitoTox database workflow and structure. **a** MitoTox is an open-access database constructed using the Django framework with a PostgreSQL database as the backend. **b** The MitoTox database structure includes entries of compounds, targets, functions and references

MitoTox is an open platform for developing web-based toxicological data discovery and reporting applications. The database is implemented in Python3 under the web framework with Django 3.0.3, the relational database with PostgreSQL, the webserver in Nginx (connected to the web framework by the Gunicorn library), and the Ubuntu system on the backend. The front end is constructed using HTML5, CSS3, and JavaScript (Fig. 1). Additional libraries, jQuery, D3.js, and Bootstrap were utilized to help us visualize the data and complete the web design. A GUI and searchable drop-down menus for the database were created by Django’s admin library and the Django-selectable library. The Django web framework basic security measures were provided by the Django web framework to restrict access to the modification of the entries of all database tables.

### Database description

**Fig. 2:**
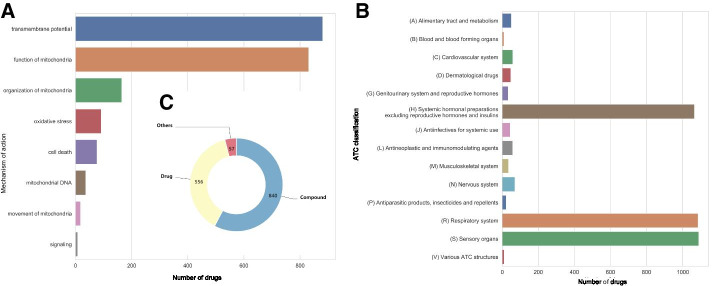
Summary and statistics of the MitoTox database. The mitochondrial toxicity-related molecules are classified according to **a** the affected mitochondrial functions, **b** the affected organ systems according to The Anatomical Therapeutic Chemical (ATC) Classification, and **c** types

MitoTox includes over 1400 small compound entries corresponding to more than 34,000 different synonyms. These compounds are linked to over 870 mitochondrial-related protein, protein subunit and RNA targets through toxin and toxin-target bonds. Figure 2 shows a summary and statistics of the MitoTox database. The toxicities are classified into 8 major groups according to their function and toxic mechanisms, into 225 functional subgroups according to their toxicity mechanisms, and into over 650 target subgroups according to their molecular targets. All the entries and relationships recorded in the database are supported by references; currently, there are approximately 300 references. One important feature of MitoTox is the storage of experimental results of in vitro and in vivo assays for the detection of drug-induced mitochondrial toxicity. The experimental results from the mitochondrial functional and toxicity assays were recorded with the dosage, corresponding experimental methods, model system, and results including IC50 (half-maximal inhibitory concentration), EC50 (half-maximal effective concentration), or Cmax (the maximum or peak serum concentration).

## Results

**Fig. 3:**
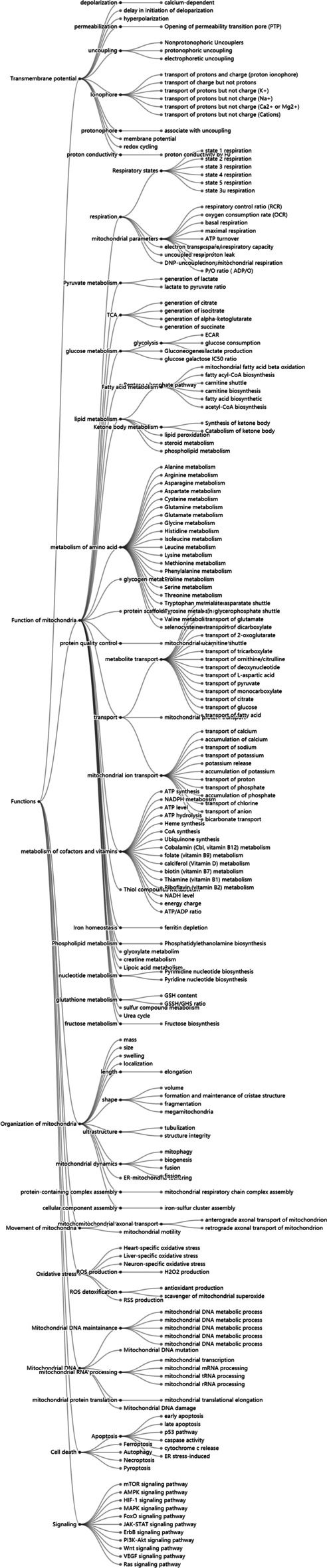
Classification of mitochondrial toxicity mechanisms according to functionality. The mechanisms of toxicity are classified into multiple layers. The first classification layer includes eight main categories in: transmembrane potential, functions of mitochondria, organization of mitochondria, movement of mitochondria, oxidative stress, mitochondrial DNA, cell death, and signaling. Each category is further classified into subclasses according to their mechanism of action

To better understand the physiological impact of each drug or compound on mitochondria, we constructed a drug database combining general pharmaceutical information and experimental data on the drugs. We searched for mitochondria-related keywords in the complete DrugBank file and found 1011 items that were possibly related to drug-induced mitochondrial toxicity. Through manual curation, we identified 180 true positive items of drug-induced mitochondrial toxicity. We applied the same pipeline to obtain drug targets, synonym names, and clinical information for these drugs. From the keyword search in PubMed, over 300 review articles and research papers reporting drugs and molecules were manually recorded in the MitoTox database. Collectively, our database currently contains 1400 drugs or compounds that have been tested for or show evidence of mitochondrial toxicity, which are classified into 77 categories of mitochondrial toxicity mechanisms (Fig. 3).

**Fig. 4:**
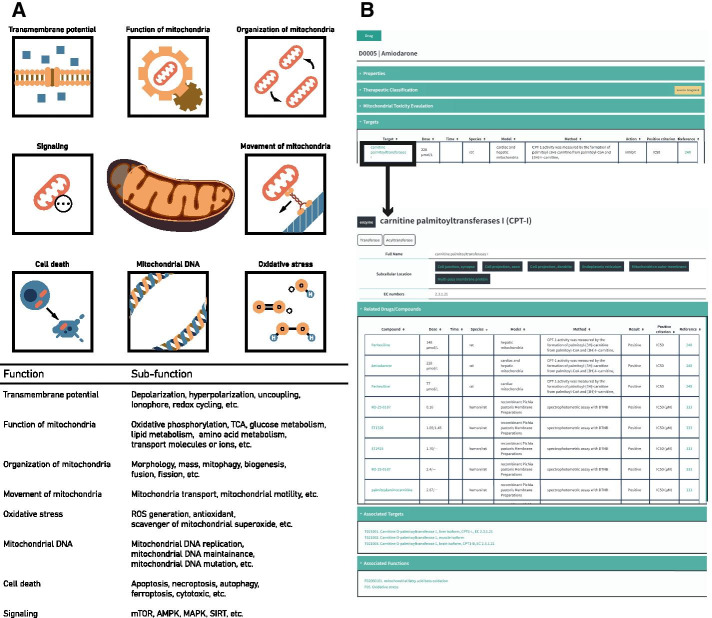
Classification of molecular targets of drugs that induce mitochondrial toxicity. **a** The molecular targets are classified into eight main categories according to the action of the targets. **b** A screenshot of the mitochondrial toxin target record using “Amiodarone” and one of its targets, “carnitine palmitoyltransferase I (CPT-1),” as an example

Mitochondrial dysfunction could stem from structural, enzymatic, and biochemical changes [28]. Mitochondrial toxicities arise from different mechanisms, including interference with the mitochondrial respiratory chain, the tricarboxylic acid (TCA) cycle, and mitochondrial DNA replication, leading to loss of mitochondrial membrane potential and increased mitochondrial oxidative stress, and eventually cell death. Based on these results, mitochondrial toxicity mechanisms are classified into eight main categories: alternation of transmembrane potential, function of mitochondria, organization of mitochondria, movement of mitochondria, oxidative stress, cell death, mitochondrial DNA, and metabolic-related signaling pathways (Fig. 4a). Each category is further classified into subgroups according to the corresponding toxicity mechanism. For example, in the category of transmembrane potential, inhibiting ETC or uncoupling or modulating transport of ions could be mechanisms that affect mitochondrial membrane potential and therefore were classified under the mitochondrial membrane potential category.

Mitochondrial toxicity-related targets are classified into mitochondrial enzymes in the matrix, transporters or carriers on the membranes, mitochondrial DNA replication machinery, RNAs, protein synthesis and assembly machinery, small molecules and metabolites, etc. The targets can also be classified based on their locations, such as the mitochondrial matrix, inner mitochondrial membrane (IMM), outer mitochondrial membrane (OMM), and intermembrane space (IMS). In each category, there are molecular targets corresponding to altering mitochondrial function and morphology. For example, in the ETC category, there are complex I, complex II, complex III, complex IV, and ATP synthase proteins. In each protein complex, there are multiple subunits or molecules that could be the targets for an individual drug. Each molecular target is recorded in the database with relevant molecular entries, such as molecular protein name and gene name, EC number, subcellular location, involved reactions, and modulators (Fig. 4b). The drugs that target mitochondria can affect multiple factors to induce mitochondrial toxicity [29]. Therefore, drug targets related to mitochondria and related metabolism or signaling pathways are cross-referenced and linked by toxin-target associations based on multiple experimental data from the literature and databases.

**Fig. 5:**
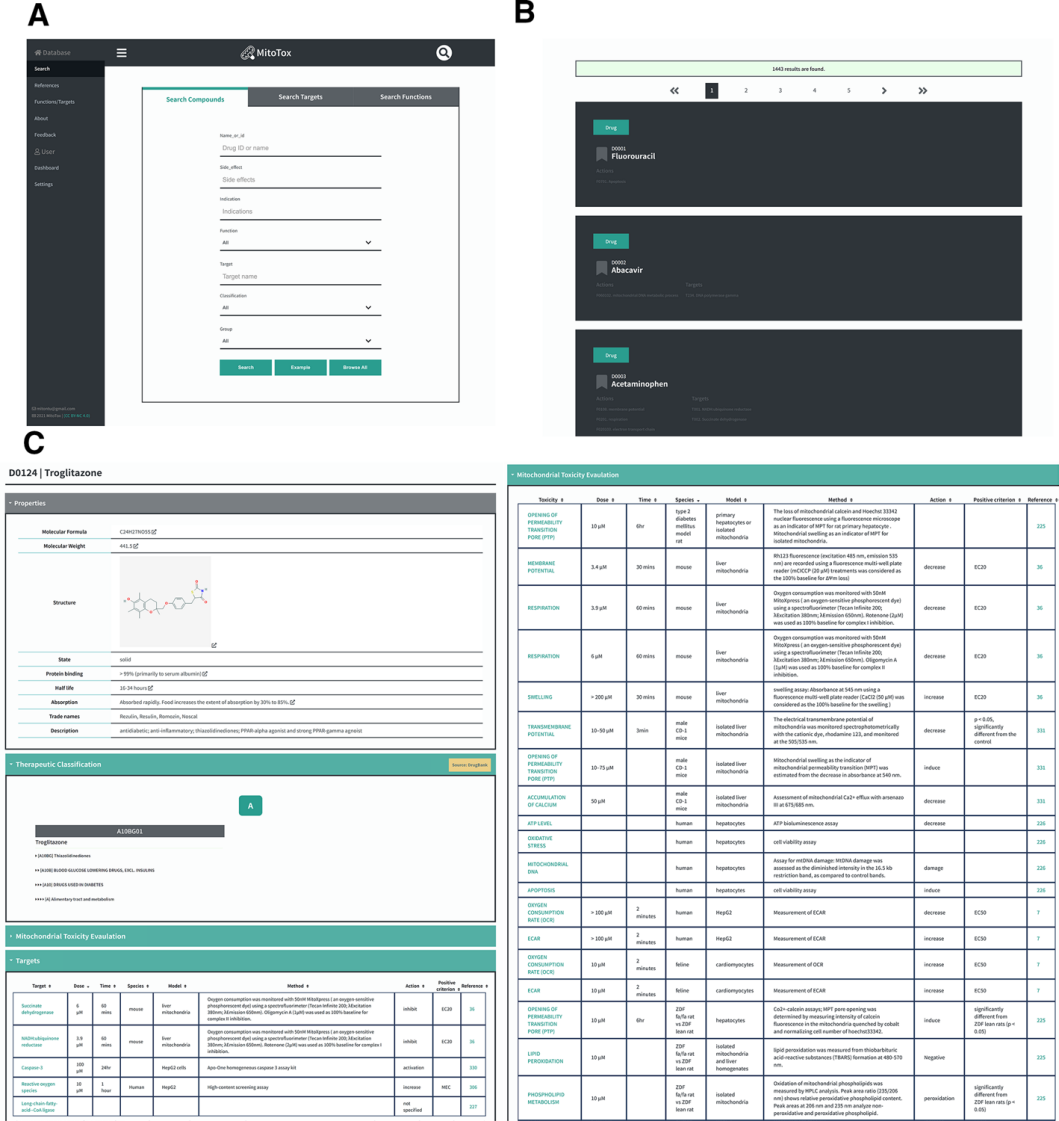
Screenshot of the MitoTox database. **a** A screenshot of the Mitochondria Toxicity Database (MitoTox) showing several search and display tools of MitoTox. **b** A screenshot of the search results. **c** Example of the data fields or data types found in MitoTox describing the mitochondrial toxin “Troglitazone.”

Common screening assays for mitochondrial toxicity include evaluation of cytotoxicity of the compounds incubated with cultured cells in glucose or galactose conditioned media (Glu/Gal assay) [30], and measurements of mitochondrial oxygen consumption [31, 32], mitochondrial membrane potential [33, 34], reactive oxygen species [31, 32], mitochondrial permeability transition [35], and cellular ATP content [21, 36, 37]. Many of these mitochondrial functional assays have been applied to high-throughput screening for mitochondrial toxicity [38]. Mitochondrial membrane potential (ΔΨm) is determined by a balance between the proton gradient and other ion gradients across the inner mitochondrial membrane maintained by proton pumps and electrogenic ion channels or transporters. Since mitochondrial membrane potential is a part of the proton-motive force that generates ATP, ΔΨm is an important indicator of mitochondrial function and cell health. Lipophilic cationic compounds (such as TMRM, TMRE, and JC1) accumulating in the mitochondrial membrane matrix space are often used to monitor ΔΨm in high-throughput screening assays. Oxygen consumption profile is another important indicator of mitochondrial function. Microplate-based assays using oxygen-sensitive fluorescent probes provide a means for high-throughput evaluation of oxygen consumption rate (OCR), reserve capacity, and extracellular acidification rate (ECAR). Drugs or compounds that significantly change the mitochondrial membrane potential and OCR profiles are included in the MitoTox database and are labeled as positive or negative entries in the assay results based on the experimental criteria. The experimental systems and conditions, including the dosage, exposure time, substrates, or growth conditions, are also recorded along with their corresponding references (Fig. 5).

## Discussion

In addition to pathological functional changes in the mitochondria, mitochondrial dysfunction resulting from drug treatment or chemical exposure is an important concern in the area of biomedical and ecological health. Mitochondrial health is essential to the survival and fitness of the organisms, and detailed investigations of mitochondrial functions are imperative. To further our understanding of the pharmacological and toxicological mechanisms influencing mitochondrial toxicity, it is necessary to have a database capable of integrating information about mitochondrial toxicity and recording data acquired by the measurements of mitochondrial functions, particularly those functions that have been perturbed by extrinsic and intrinsic factors. Many assays have been developed and implemented to investigate the functional and morphological changes in mitochondria. Respiration rate and ATP content are important indicators of mitochondrial health. Mitochondrial stress can be assessed based on the parameters of ROS production, mitochondrial dynamics, permeability transition, and quality control. In particular, oxidative stress is an important issue in mitochondrial toxicity. ROS are the side-products of mitochondrial electron transfer and are regulators of cellular signaling during growth, differentiation, and metabolism in low physiological doses; however, excessive levels of ROS cause oxidative damage and cell death. Nevertheless, many chemical compounds and environmental pollutants are involved in the generation of reactive species and oxidative stress, thus influencing mitochondrial and cellular functions [32, 39, 40].

An electronic database that provides a searchable index and keywords is constructed and presented in this paper to facilitate drug safety and toxicity prevention. By collecting such data, we could examine the association between physiological responses, such as membrane potential changes and ATP production, and known drug toxicity and clinical side effects. These data should produce a repository of useful information for future mitochondrial toxicity studies, build an algorithm for mitochondrial toxicity prediction, and shed light on candidate drug selection during drug discovery.

Development of high-throughput technologies increased availability and accessibility of mitochondrial toxicity-related data. Big data containing mitochondria-related information obtained from high-throughput or high-content systems provide opportunities for statistical analysis and machine learning to classify the mechanism of action and discover or predict potential mitochondrial toxicants and therapeutics. Statistical methods, such as Fisher’s exact test, can be used to define associations between the mechanisms of mitochondrial toxicity and side effects. In addition to enhanced insight into mitochondrial toxicity, the recent rapid development of machine learning technologies can be used to analyze and predict side effects [41, 42] or identify toxicophores that are predictive of mitochondrial toxicity [43].

Our purpose in building MitoTox is to study how drug-induced mitochondrial toxicity causes side effects in clinical research. The MitoTox database bridges the gap between the two sides (mitochondrial toxicity vs. side effects) and could help predict potential mitochondrial toxicity for future drug discovery. The combined clinical information, including clinical indications, side effects, frequency of side effects, and therapeutic indications with molecular toxicity mechanisms, enables us to learn and prevent potential side effects.

## Conclusion

MitoTox database is intended to provide novel strategies for the preclinical screening of mitochondrial toxicity, which will be of immediate benefit to drug safety and global health. Furthermore, the planned investigations of the mitochondrial toxicity subtypes are expected to elucidate how the morphological features and functional indicators of mitochondria may serve as a pathological feature of disease onset and disease progression. MitoTox will help advance our understanding of mitochondrial-associated toxicity and provide new possibilities for the early diagnosis of mitochondrial diseases, as well as innovative therapeutic strategies against these diseases. Last but not least, understanding the mitochondrial toxicity of drugs could help in the development of drugs that target mitochondria to treat common pathologies [44].

## Data Availability

MitoTox is an open access database at http://www.mitotox.org.

## Abbreviations

ATP: Adenosine triphosphate
MPTP: 1-Methyl-4-phenyl-1, 2, 3, 6-tetrahydropyridine
ΔΨm: Mitochondrial membrane potential
TCA cycle: Tricarboxylic acid cycle
IMM: Inner mitochondrial membrane
OMM: Outer mitochondrial membrane
IMS: Intermembrane space
OCR: Oxygen consumption rate
ECAR: Extracellular acidification rate
